# When a virus lies in wait

**DOI:** 10.7554/eLife.71121

**Published:** 2021-08-04

**Authors:** David Taussig, Yariv Wine

**Affiliations:** The Shmunis School of Biomedicine and Cancer Research, the George S. Wise Faculty of Life Sciences, Tel Aviv University Tel Aviv Israel

**Keywords:** arthritis, gammaherpesvirus-68, Epstein-Barr virus, age-associated B cells, latent infection, Mouse, Virus

## Abstract

A mouse model supports the hypothesis that latent Epstein–Barr virus exacerbates the symptoms of rheumatoid arthritis.

**Related research article** Mouat IC, Morse ZJ, Shanina I, Brown KL, Horwitz MS. 2021. Latent gammaherpesvirus exacerbates arthritis through modification of age-associated B cells. *eLife* **10**:e67024. doi: 10.7554/eLife.67024

The body’s ability to fight a wide range of external (e.g. viruses, bacteria) and internal (e.g. cancer cells) threats relies on having a fully functional immune system. The immune system can be roughly divided into two parts: the innate system, which is activated quickly and considered the first line of defense; and the adaptive system, which usually ‘kicks in’ a few days following the initial infection and evolves over time to generate a long-lasting and specific response (Murphy et al., 2017).

The power of the adaptive immune system stems from the ability of its white blood cells – T cells and B cells – to express a diverse set of immune receptors that can bind to specific pathogens (Rubelt et al., 2017). As well as having receptors bound to their surface, B cells also secrete some of their receptors in the form of antibodies.

Some B cells, however, produce ‘autoantibodies’ that recognize and bind to molecules on the body’s own cells, causing the immune system to mistakenly attack normal, healthy tissue (Sogkas et al., 2021). There are many mechanisms in place to eliminate these B cells, but if they fail autoimmune disorders can arise (Theofilopoulos et al., 2017). Autoimmune diseases, such as systemic lupus erythematosus multiple sclerosis and rheumatoid arthritis, are thought to be triggered by past viral infections (McClain et al., 2005; Mameli et al., 2012; Costenbader and Karlson, 2006). For example, rheumatoid arthritis (RA) – an inflammatory autoimmune disease that affects the joints – has been associated with Epstein-Barr virus (EBV) infection (Guo et al., 2018; Costenbader and Karlson, 2006).

Most exposures to EBV occur during childhood or adolescence without leading to RA, but the disease tends to appear after patients turn 60. Thus, it is hypothesized that EBV latency – a situation in which the virus becomes dormant within the host cell without producing more viruses – may contribute to RA symptoms appearing later in life. This is in agreement with a previous study in which mice with an autoimmune condition called experimental autoimmune encephalopathy were latently infected with a homolog of EBV called gammaherpesvirus 68 (γHV68). The infection exacerbated the autoimmune condition without changing autoantibody levels, and led to a disease that resembles multiple sclerosis (Casiraghi et al., 2012). But it is unclear whether EBV has the same effect on RA.

Now, in eLife, Marc Horwitz and colleagues from the University of British Columbia – including Isobel Mouat as first author – report that latent viral infection also worsens the symptoms of a mouse model for RA (Mouat et al., 2021). They used a mouse model for RA that suffers from an autoimmune condition called type II collagen-induced arthritis (CIA). When these mice are exposed to type II collagen, they develop arthritis with a sustained T cell response and as a result produce anti-collagen autoantibodies that can infiltrate the joints (Inglis et al., 2007).

Mouat et al. infected CIA mice with latent γHV68 to develop a mouse model that can shed light on how EBV may affect the symptoms of RA in humans. The experiments showed that infection increased the clinical parameters used to measure the severity of CIA (Figure 1), demonstrating the negative impact of the latent virus. Specifically, a subset of B cells called age-associated B cells were shown to be expressed in significantly higher proportions than in uninfected mice. Finally, Mouat et al. used a strain of γHV68 that cannot become latent to show that latency is necessary for the virus to exacerbate CIA.

**Figure 1.**
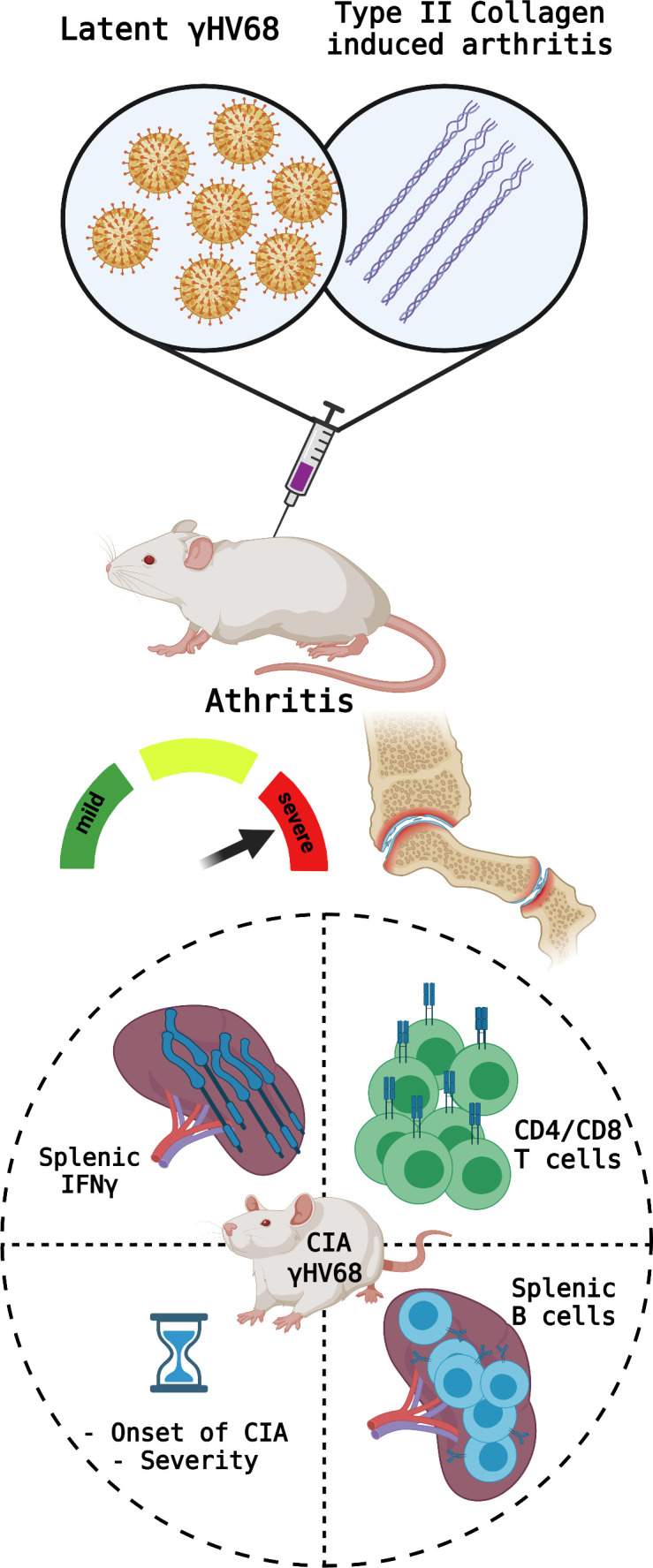
Parameters measured to determine CIA severity. Top: Mice were infected with latent γHV68 and treated with type II collagen to model the effects of Epstein-Barr virus on rheumatoid arthritis. This manipulation causes the symptoms of type II collagen arthritis (CIA) to become more severe and increase inflammation in the mice’s joints. Bottom: the parameters measured to assess the severity of the autoimmune disease include (circle, clockwise from top left): the levels of the cytokine IFNγ in the spleen (which increased in the CIA mice infected with γHV68); the proportions of different T cell populations (which became similar to what is observed during infections); the phenotypes of B cells in the spleen (more age-associated B cells were observed); and the time until onset of the disease (which decreased). Created with BioRender.com.

Mouat et al. show that latent γHV68 infection in mice clearly alters autoimmune disease onset and severity, potentially reflecting how latent EBV infection affects RA in humans. Future research will be needed to reveal exactly how B cells are impacted by EBV. Notably, the development and validation of the RA model in mice will facilitate future studies aiming to better understand the nature of the relationship between EBV infection and RA.
